# Clinical Correlates of Mass Effect in Autosomal Dominant Polycystic Kidney Disease

**DOI:** 10.1371/journal.pone.0144526

**Published:** 2015-12-07

**Authors:** Hyunsuk Kim, Hayne Cho Park, Hyunjin Ryu, Kiwon Kim, Hyo Sang Kim, Kook-Hwan Oh, Su Jong Yu, Jin Wook Chung, Jeong Yeon Cho, Seung Hyup Kim, Hae Il Cheong, Kyubeck Lee, Jong Hoon Park, York Pei, Young-Hwan Hwang, Curie Ahn

**Affiliations:** 1 Department of Internal Medicine, Seoul National University Hospital, Seoul, Korea; 2 Department of Internal Medicine, Armed Forces Capital Hospital, Seongnam-si, Gyeonggi-do, Korea; 3 Nephrology Clinic, National Cancer Center, Goyang-si, Gyeonggi-do, Korea; 4 Department of Internal Medicine, Asan Medical Center, University of Ulsan, Seoul, Korea; 5 Division of Hepatology, Seoul National University Hospital, Seoul, Korea; 6 Department of Radiology, Seoul National University Hospital, Seoul, Korea; 7 Department of Pediatrics, Seoul National University Children’s Hospital, Seoul, Korea; 8 Research Coordination Center for Rare Diseases, Seoul National University Hospital, Seoul, Korea; 9 Department of Internal Medicine, Kangbuk Samsung Medical Center, Seoul, Korea; 10 Department of Biological Science, Sookmyoung Women’s University, Seoul, Korea; 11 Division of Nephrology, Department of Internal Medicine, University Health Network and University of Toronto, Ontario, Canada; 12 Department of Internal Medicine, Eulji General Hospital, Seoul, Korea; University of Bari Aldo Moro, ITALY

## Abstract

Mass effect from polycystic kidney and liver enlargement can result in significant clinical complications and symptoms in autosomal dominant polycystic kidney disease (ADPKD). In this single-center study, we examined the correlation of height-adjusted total liver volume (htTLV) and total kidney volume (htTKV) by CT imaging with hepatic complications (n = 461) and abdominal symptoms (n = 253) in patients with ADPKD. “Mass-effect” complications were assessed by review of medical records and abdominal symptoms, by a standardized research questionnaire. Overall, 91.8% of patients had 4 or more liver cysts on CT scans. Polycystic liver disease (PLD) was classified as none or mild (htTLV < 1,600 mL/m); moderate (1,600 ≤ htTLV <3,200 mL/m); and severe (htTLV ≥ 3,200 mL/m). The prevalence of moderate and severe PLD in our patient cohort was 11.7% (*n* = 54/461) and 4.8% (*n* = 22/461), respectively, with a female predominance in both the moderate (61.1%) and severe (95.5%) PLD groups. Pressure-related complications such as leg edema (20.4%), ascites (16.6%), and hernia (3.6%) were common, and patients with moderate to severe PLD exhibited a 6-fold increased risk (compared to no or mild PLD) for these complications in multivariate analysis. Similarly, abdominal symptoms including back pain (58.8%), flank pain (53.1%), abdominal fullness (46.5%), and dyspnea/chest-discomfort (44.3%) were very common, and patients with moderate to severe PLD exhibited a 5-fold increased risk for these symptoms. Moderate to severe PLD is a common and clinically important problem in ~16% of patients with ADPKD who may benefit from referral to specialized centers for further management.

## Introduction

Development of liver cysts is the most common extra-renal manifestation of autosomal dominant polycystic kidney disease (ADPKD) and occurs with increasing age [1,2]. In the multi-center Consortium for Radiological Imaging Studies of Polycystic Kidney Disease (CRISP), the prevalence of liver cysts by magnetic resonance imaging (MRI) was 58% in patients aged 15–24 years, 85% in patients aged 25–34 years, and 94% in patients older than 35 years [3]. Among those patients with liver cysts, a subset of them with significant liver cystic burden will experience polycystic liver disease (PLD) with its associated clinical symptoms (e.g. abdominal fullness or pain, early satiety, dyspnea) and “pressure-related” complications (e.g. leg edema, ascites, portal hypertension, abdominal hernia) [1,2]. In contrast to polycystic kidney disease, PLD rarely causes hepatic failure but can result in significant morbidities from its “mass-effect” [4–6]. In the HALT-PKD-A clinical trial, the prevalence of moderate (height adjust liver volume or htLV = 1,000–1,800 mL/m) and severe (htLV >1,800 mL/m) PLD by MRI was reported to be ~50% and 5%, respectively [7]. However, the prevalence and clinical correlates of PLD have not been well defined, especially in a large patient cohort from a single geographical region. Moreover, the “mass effect” symptoms and complications in patients with PLD can be further aggravated by their enlarged kidneys. In the current study, we present our systematic study of “mass-effect” from liver and kidney enlargement in a large cohort of well-characterized patients from a regional ADPKD clinical center in Seoul, Korea.

## Materials and Methods

### Study patients

We reviewed 488 patients older than 20 years of age who were registered at the ADPKD clinic of Seoul National University Hospital in Seoul, Korea, between October 2009 and September 2012. ADPKD was diagnosed according to the unified criteria [8]. Among these cases, 27 were excluded because of active treatments for extra-hepatic malignancies (n = 2), cancer with liver metastasis (n = 1), hepatocellular cancer with previous partial hepatectomy (n = 1), liver cirrhosis (n = 4), chronic HBV or HCV hepatitis (n = 18), and hepatectomy due to IHD stone (n = 1). The remaining 461 cases were included for the analyses (S1 Fig). For clinical symptom assessment, 253 patients who completed a research questionnaire between May 2013 to September 2013 were analyzed (S3 Table); and 195 patients who refused or were unavailable to complete the questionnaire and 13 who had prior PLD therapeutic interventions were excluded. Review of medical records, however, was performed in all 461 patients to ascertain their PLD-related complications. This study was approved by the Institutional Review Board at Seoul National University Hospital (H-1002-028-309) and performed in accordance with the Declaration of Helsinki. All patients provided written informed consent prior to participation.

### Clinical assessment

The following baseline data were collected in all 461 study patients: baseline demographic profiles, medical history of hypertension, cancer, and chronic liver disease, and laboratory data including serum albumin, alkaline phosphatase (ALP), aspartate aminotransferase (AST), alanine aminotransferase (ALT), total cholesterol (TC), and serum creatinine (Cr). They also had computed tomography (CT) which were performed biennially in all our clinic patients using multi-detector CT scanners. The presence or absence of 4 or more hepatic cysts were defined manually by one skilled technician based on review of each CT scan [6, 9].

Hypertension was defined as blood pressure > 140/90 mmHg or a history of treatment with antihypertensive medications. Chronic liver disease was defined as chronic hepatitis (toxic, alcoholic, viral, or autoimmune) or liver cirrhosis. Serum Cr was measured using the Jaffe method, traceable using isotope dilution mass spectrometry (IDMS). The Chronic Kidney Disease Epidemiology Collaboration Formula (CKD-EPI) was used to calculate the estimated glomerular filtration rate (eGFR) [10]. CKD stages were classified according to the National Kidney Foundation’s Kidney Disease Outcomes Quality Initiative (K/DOQI) guidelines [11]. End-stage renal disease (ESRD) was defined as an eGFR < 15 mL•min−1•1.73 m−2 or initiation of renal replacement therapy. Intervention was defined as trans-arterial embolization, partial hepatectomy, or liver transplantation.

### Measurements of liver and kidney volumes

Total liver volume (TLV) was measured by stereology and manual segmentation using Rapidia 2.8 CT software (INFINITT, Seoul, Korea) [12], which calculates the total volume from a set of contiguous images by summing the products of area and slice thickness of CT scan image. Large vascular structures such as the IVC and portal veins were excluded from the TLV. Total kidney volume (TKV) was calculated by the modified ellipsoid method which has been shown to strongly correlated with the standard method by stereology (R^2^~0.98) [13], Height-adjusted TLV (htTLV) and TKV (htTKV) were used for analysis.

### Investigation of hepatic complications

Medical records were reviewed retrospectively with hepatic complications divided into three categories: pressure-related (i.e. ascites, hernia, bilateral leg edema, biliary dilatation, or inferior vena cava [IVC] stenosis), infections (i.e. liver cyst infections or cholangitis), and others (i.e. splenomegaly or Caroli disease). Ascites were detected by CT scan and classified as: small, minimal layer of ascites in the gravity-dependent regions of the peritoneal and/or perihepatic cavity; moderate, presence of fluid in the paracolic gutters; and large, sufficient ascites to displace the small intestinal loops. Cyst infection was defined as tenderness on a cystic lesion, signs of infection, and positive findings on imaging studies, and/or aspiration of infected cysts [14]. Cyst rupture was defined as disrupted cyst wall on CT with compatible history. Biliary dilatation was defined as abnormal extrahepatic or intrahepatic dilatation due to hepatic cyst compression by CT. Cholangitis was defined as the presence of fever, abdominal pain, direct hyperbilirubinemia, and/or positive blood culture. Splenomegaly was defined as the largest dimension of the spleen being >11 cm. IVC stenosis was defined as stenosis or obliteration due to pressure or thrombosis and IVC stent insertion.

### Investigation of mass-effect symptoms

Symptoms were assessed with a questionnaire that was generated from the Gastrointestinal Symptom Rating Scale (GSRS) with minor modifications (S3 Table) [15]. The questionnaire had 11 questions in Korean that measured symptoms graded from 0–3 points, except for the question about the sense of a mass (0 or 1). The patients completed the questionnaire in the presence of an interviewer. The questions were grouped according to symptoms related to pressure (early satiety, dyspnea or chest discomfort, palpable mass, and abdominal fullness), pain (right upper quadrant [RUQ] pain or discomfort, flank pain or discomfort, back pain or discomfort, and intake of analgesics), and the GI tract (nausea or vomiting, anorexia, and epigastric soreness).

### Statistical analysis

The prevalence of liver cysts and distribution of htTLV were analyzed by the chi-square test, Fisher’s exact test, the Mann–Whitney U-test, and Jonckheere–Terpstra test, where appropriate. Log odds graphs of complications or symptoms according to htTLV were plotted using R statistical software (R Foundation for Statistical Computing, Vienna, Austria, http://www.R-project.org/). The association of clinical characteristics with htTLV was analyzed by linear by linear association test. Furthermore, the prevalence of symptoms and complications were compared by linear association test or Jonckheere–Terpstra test, where appropriate. Multivariate binominal logistic regression analysis of symptoms and complications was performed, adjusted for variables showing a possible association (P<0.1) in the univariate analysis. The data were analyzed by SPSS version 21.0 (IBM SPSS, Armonk, NY). All reported P values are two-tailed, and the statistical significance threshold was set at P < 0.05.

## Results

### Baseline characteristics

The clinical characteristics of the study patients (n = 461) with TKV, TLV, and retrospective review of “mass-effect” complications are shown in S1 Table. Their mean (± SD) age on Oct, 2013 was 51 ± 13 years and 52.3% (241/461) were female. Their mean (± SD) estimated glomerular filtration rate (eGFR) was 80.0 ± 27.7 mL•min^−1^•1.73 m^−2^. The prevalence of hypertension in this cohort was 79.2%. Their median [interquartile range (IQR)] htTKV and htTLV was 820 [453, 1345] and 986 [825, 1,280] mL/m, respectively. The clinical characteristics of a subset of this cohort (n = 253) who completed a clinical symptom questionnaire are also shown in S1 Table. In general, the mean age, gender, mean eGFR, prevalence of hypertension, and both htTKV and htTLV did not differ between the two groups, except for a high prevalence of CKD stage 3 and lower prevalence in CKD stage 5 in the latter.

### Prevalence of liver cysts and polycystic liver disease

**Fig 1:**
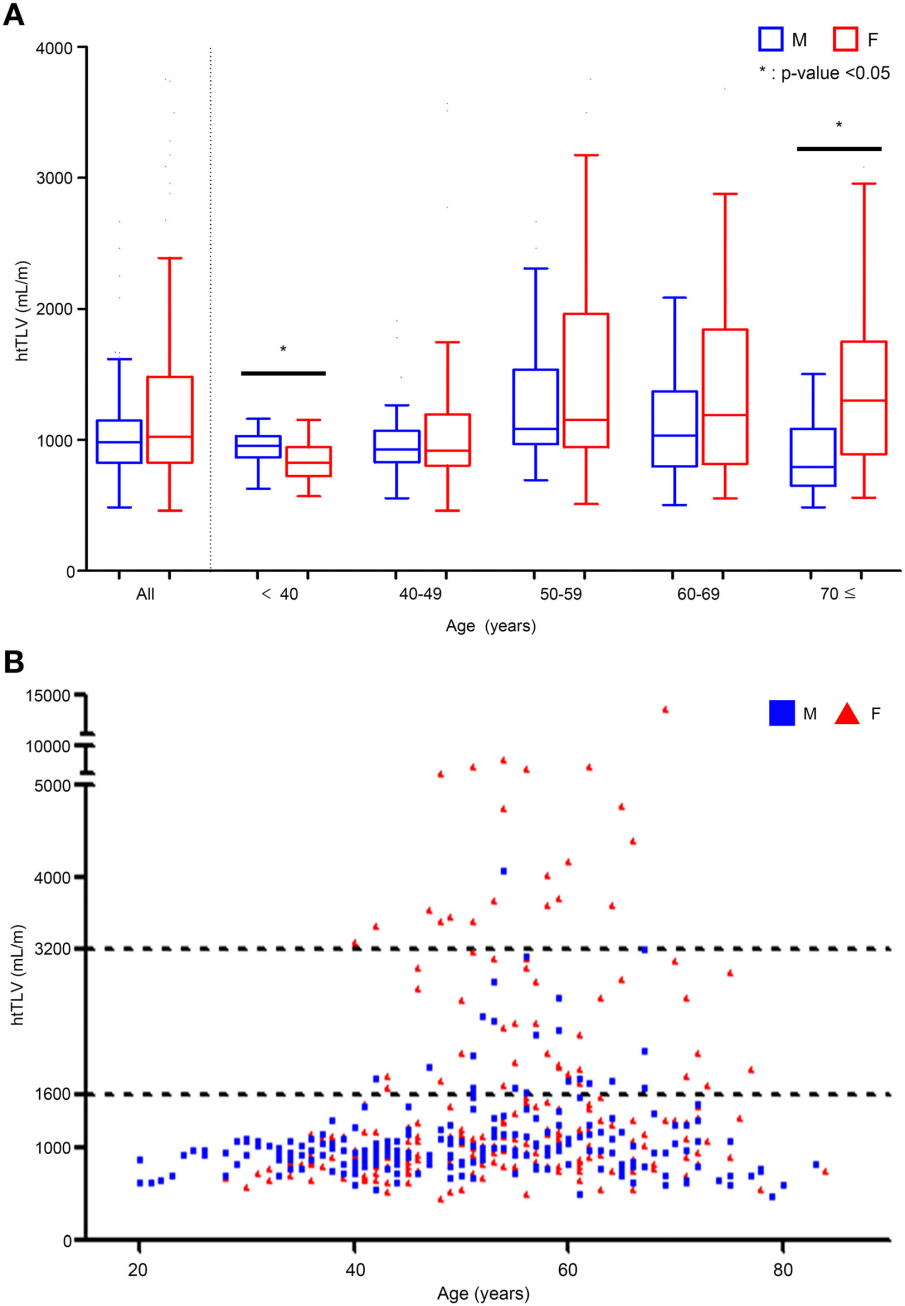
Distribution of height-adjusted total liver volume (htTLV) according to age and gender. (A) Box plot of median (and inter-quartile range) of htTLV by age decades. The median htTLV was higher in males of age <40 and females ≥70 years (*P < 0.05 for gender difference). The skewing of htTLV towards larger size is most noticeable in females across all strata above 40 years of age, (*P* value for Jonckheere–Terpstra test for trend <0.001). (B) A scatter plot of htTLV by age and gender. Moderate PLD is moderately enriched by females and severe PLD, extremely enriched by females.

91.8% (423/461) of study patients had 4 or more liver cysts by CT scan with a higher prevalence of liver cysts (P < 0.001) in females (96.2% or 232/241) than males (% or 191/220), especially in females (96.8% or 30/31) than males (74% or 37/50) under 40 years of age. Fig 1 shows the distribution of TLV of the study cohort by their gender and age strata. Under 40 years of age, the median of htTLV was larger in male than female patients (955 *vs*. 823 mL/m, *P* = 0.017, Fig 1A). Although the median htTLV did not differ in most age strata between the two gender, females displayed a highly skewed distribution towards larger htTLV compared to males above 40 years of age (*P* for trend < 0.001 by Jonckheere–Terpstra test, Fig 1A). Females older than 70 years also had larger median htTLV than males (1,300 *vs*. 791 mL/m, *P* = 0.005, Fig 1A). The average TLV of deceased liver donors was close to 1,400mL in Koreans [16], which can be translated into mean htTLV of ~850 mL/m. Therefore, htTLV values of 1,600, and 3,200 mL/m correspond to approximately two-, and four-times of normal htTLV, respectively. We classified all patients into three groups according to their htTLV: (i) no or mild PLD, htTLV < 1,600 mL/m; (ii) moderate PLD, 1,600 ≤ htTLV <3,200mL/m; and (iii) severe PLD, htTLV ≥ 3,200 mL/m. The proportion of no or mild, moderate, and severe PLD was 83.5% (*n* = 385), 11.7% (*n* = 54), and 4.8% (*n* = 22) (S2 Table). Fig 1B shows that moderate (1,600 mL≤htTLV<3,200 mL) and severe (3,200 mL<htTLV) PLD typically occurred in patients older than 40 years. There was a significant female predominance (female:male = 33:21, *P*<0.001) for moderate PLD and an extreme female predominance (female:male = 21:1, *P*<0.001) for severe PLD, which account for the highly skewed distribution of htTLV in female patients seen in Fig 1A.

### Relationship of hepatic complications and abdominal symptoms with htTLV

**Fig 2:**
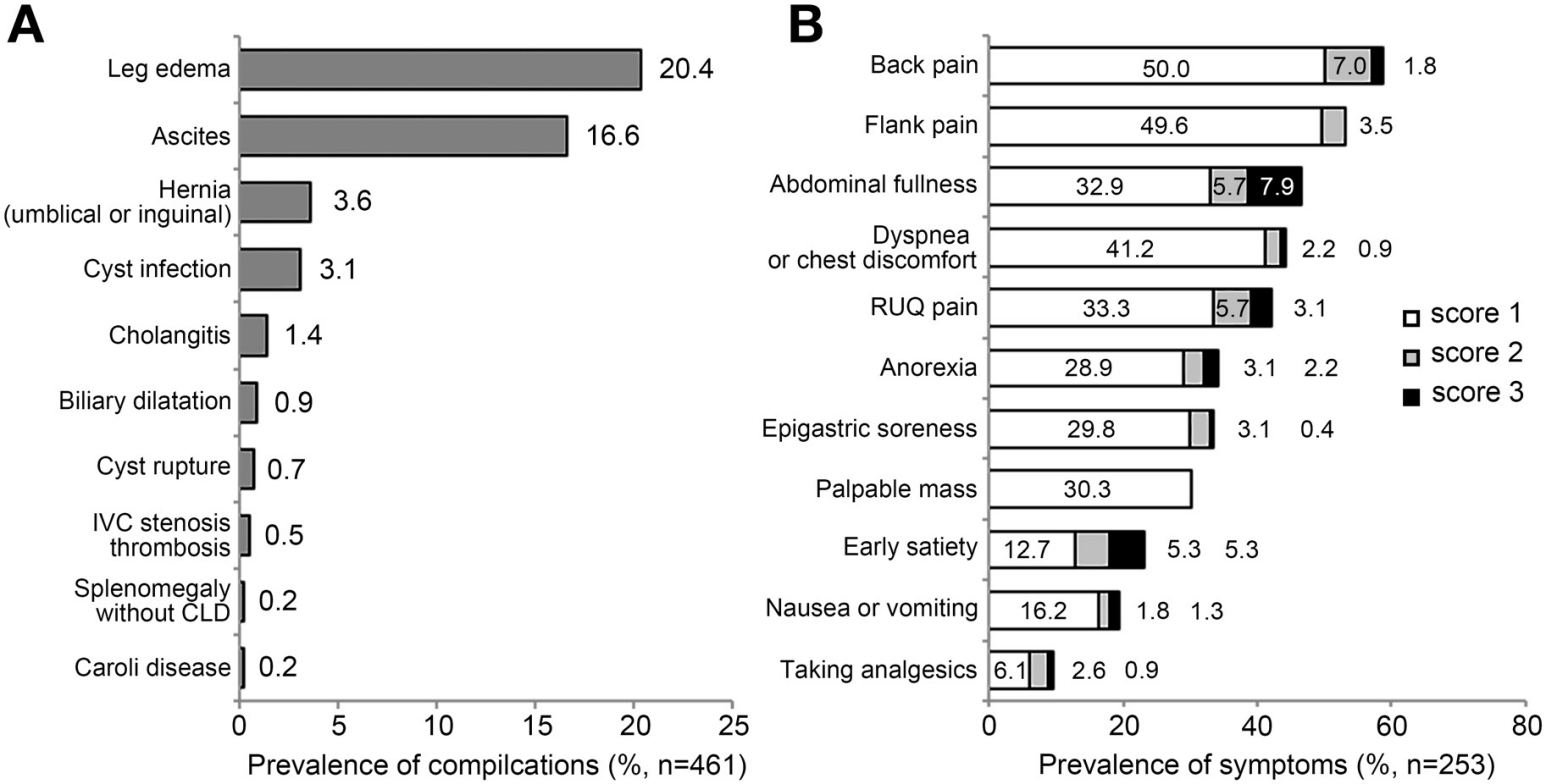
Prevalence of abdominal symptoms and hepatic complications. (A) The prevalence of hepatic complications of all subjects. (B) The prevalence of abdominal symptoms on a three-point scale. Back pain and flank pain were most prevalent. Abdominal fullness and early satiety were common among moderate to severe symptoms (point 2 or 3).

Among the complete cohort (n = 461), the most common complication was leg edema (20.4%), followed by ascites (16.6%), hernia (3.6%), and cyst infection (3.1%) (Fig 2A). In the subset of 253 patients who completed the clinical symptoms questionnaire, the most common pressure-related symptoms were back pain (58.8%) and flank pain (53.1%), followed by abdominal fullness (46.5%), dyspnea or chest discomfort (44.3%), mass sensation (30.3%), and early satiety (23.3%). The most common moderate to severe (score 2 or 3) pressure-related symptoms was abdominal fullness (13.6%), followed by early satiety (10.6%), back pain and right upper quadrant pain (both 8.8%) (Fig 2B).

**Fig 3:**
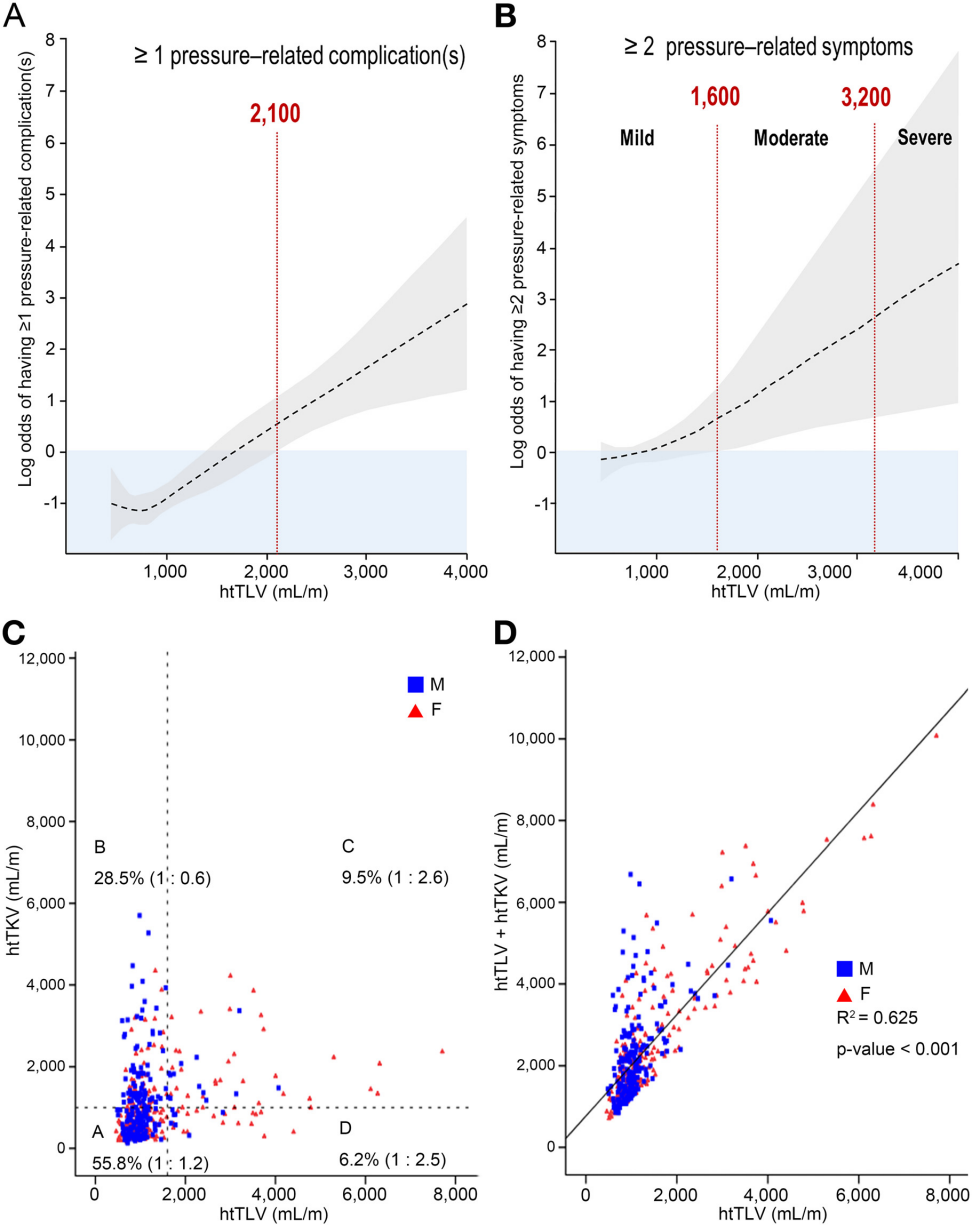
Likelihood (log odds) of having complications or two or more pressure-related symptoms according to htTLV and the correlation between htTLV and htTKV or htTLV + htTKV. (A) Positive likelihood of having pressure-related complications at htTLV ≥2,100 mL/m. (B) Positive likelihood of having two or more pressure–related symptoms at htTLV ≥ 1,600 mL/m. Note that htTLV≥3,200 mL/m was indicated as the threshold for severe polycystic liver disease. (C) The proportion of subjects and ratio of male to female according to htTLV of 1,600 mL/m and htTKV of 1,000 mL/m. (D) Correlation between htTLV and htTLV + htTKV.

As noted earlier, there was a female predominance with increased severity of PLD (no or mild vs. moderate vs. severe PLD: 48.6% vs. 61.1% vs. 95.5%, respectively) and almost all patients with severe PLD were females. S2 Table shows that there was also a trend for older patient age (50.2 *vs*. 57.8 vs. 54.9 years, *P* for trend < 0.001), lower mean eGFR (74 *vs*. 55 vs. 63 mL·min^−1^·1.73 m^−2^, *P*<0.001), lower albumin level (4.3 *vs*. 4.0 vs. 3.8 g/dL, *P*<0.001), and lower total cholesterol (178 *vs*. 160 vs. 155 mg/dL, *P*<0.001), and higher frequency of therapeutic interventions (0 vs. 13.0% vs. 36.4%, *P* < 0.001) with increased severity (no or mild vs. moderate vs. severe) of PLD. Fig 3A and 3B show a positive correlation of the mean (95% confidence intervals) log odds of having one or more pressure-related complications and 2 or more symptoms with increasing htTLV.

Pressure-related complications such as leg edema (no or mild vs. moderate vs. severe PLD: 15.3% vs. 46.3% vs. 71.4%; *P*<0.001), ascites (10.6% vs. 38.9% vs. 54.5%; *P*<0.001), and hernia (2.9% vs. 25.9% vs. 35.7%; *P*<0.001) were significantly more common with increasing severity of PLD (all *P* <0.003, Table 1). The difference in the prevalence of leg edema was also grew up with increasing severity of PLD among subjects with eGFR ≥ 30 mL•min^−1^•1.73 m^−2^ (16% *vs*. 51.7% vs. 54.5%, *P*<0.001). When the complications were classified into pressure-related, infection-associated, or others, the frequency of pressure-related and infection-associated complications increased significantly with increasing PLD severity (*P* for trend<0.001) (Fig A in S2 Fig).

**Table 1:** Prevalence of hepatic complications according to no or mild, moderate, and severe PLD.

	No or mild	Moderate	Severe	P value ^a^
**Subjects of complication analysis (n)**	385	54	22	
**Pressure-related complications (%)**				
Leg edema	15.3	46.3	71.4	<0.001
Ascites	10.6	38.9	54.5	<0.001
Hernia	2.9	25.9	35.7	<0.001
Cyst rupture	0.3	1.9	9.1	<0.001
IVC stenosis or thrombosis	0.3	2.7	4.5	0.002
Biliary dilatation	0.5	5.6	4.5	0.003
**Infection (%)**				
Cyst infection	1.6	11.1	18.2	<0.001
Cholangitis	0	5.6	4.5	<0.001
**Others (%)**				
Splenomegaly without CLD	0.2	0	0	0.678
Caroli disease	0.2	0	0	0.678

^a^ Chi-square test for trend; None or mild PLD, htTLV<1,600 mL/m; Moderate PLD, 1,600≤ htTLV <3,200mL/m; Severe PLD, htTLV≥3,200 mL/m; PLD, polycystic liver disease; IVC, inferior vena cava; CLD, chronic liver disease.

Pressure-related symptoms such as abdominal fullness (no or mild vs. moderate vs. severe PLD: 34.8% vs. 76.3% vs. 92.9%; *P* <0.001), mass sensation (15.4% vs. 73.7% vs. 85.7%; *P* <0.001), dyspnea or chest discomfort (35.8% vs. 55.3% vs. 64.3%; *P* = 0.004), and early satiety (12.9% vs. 52.6% vs. 78.6%; *P*<0.001) were significantly more common with increasing PLD severity (Table 2). Except for flank pain, other symptoms including pain and GI symptoms increased also with increasing htTLV (*P*<0.05). We analyzed the prevalence and mean severity scores of pressure-related symptoms, pain, and GI symptoms. With increasing htTLV, there was an increase in the prevalence of pressure-related symptoms (prevalence, 57.2% *vs*. 94.7% vs. 100%, *P*<0.001), pain (69.5% vs.92.1% *vs*. 82.9%, *P*<0.001), and GI symptoms (47.0% vs.78.9% *vs*. 85.7%, *P*<0.001). Similarly, with increasing htTLV there was an increase in the mean severity scores (standardized on the basis of 100 points for comparison) for pressure-related symptoms (11 vs. 41 vs. 56, *P*<0.001), for pain (12 vs. 27 vs. 31, *P*<0.001), and for GI symptoms (9 vs. 28 vs. 32, *P*<0.001) (Fig B in S2 Fig).

**Table 2:** Prevalence of abdominal symptoms according to PLD disease severity.

	No or mild	Moderate	Severe	P value ^a^
**Responder of symptom questionnaire(n)**	201	38	14	
**Pressure-related symptoms (%)**				
Abdominal fullness	34.8	76.3	92.9	<0.001
Mass sensation	15.4	73.7	85.7	<0.001
Dyspnea or chest discomfort	35.8	55.3	64.3	0.004
Early satiety	12.9	52.6	78.6	<0.001
**Pain (%)**				
RUQ pain	30.8	68.4	92.9	<0.001
Back pain	52.0	63.2	78.6	0.028
Flank pain	48.3	63.2	50.0	0.294
Taking analgesics	5.5	23.7	21.4	<0.001
**GI symptoms (%)**				
Epigastic soreness	27.9	47.4	57.1	0.025
Anorexia	29.0	39.5	57.1	0.019
Nausea or vomiting	13.9	31.6	35.7	0.002
**Presence of** * **moderate to severe symptoms**				
**Pressure-related symptoms (%)**				
Abdominal fullness	3.0	42.1	78.6	<0.001
Early satiety	3.0	39.5	50.0	<0.001
Dyspnea or chest discomfort	1.0	10.5	14.3	<0.001
**Pain (%)**				
RUQ pain	3.0	26.3	50.0	<0.001
Back pain	3.0	26.3	35.7	<0.001
Flank pain	2.0	10.5	7.1	0.025
Taking analgesics	0.5	15.8	7.1	<0.001
**GI symptoms (%)**				
Anorexia	1.5	18.4	21.4	<0.001
Epigastic soreness	1.0	15.8	0.0	0.011
Nausea or vomiting	1.7	7.9	14.3	0.001

^a^ Chi-square test for trend; None or mild PLD, htTLV<1,600 mL/m; Moderate PLD, 1,600≤ htTLV <3,200mL/m; Severe PLD, htTLV≥3,200 mL/m;

* Moderate to severe symptoms: score 2 or 3.

PLD, polycystic liver disease; RUQ, right upper quadrant, GI, gastrointestinal.

### Height-adjusted TLV as a predictor for hepatic complications and abdominal symptoms

We tested and found htTLV as an independent risk factor for hepatic complications by multivariate logistic regression analysis, adjusting for age, gender, log (htTKV), CKD stages, albumin, total cholesterol, aspartate aminotransferase (AST), and alkaline phosphatase (ALP) (Table 3). Patients with moderate to severe PLD (htTLV≥1,600 mL/m) showed a 6-fold higher risk of hepatic complications (odds ratio [OR] 6.14, 95% confidence interval [CI]: 2.97–12.72). In a sub-group analysis of complications, htTLV was significantly associated with pressure-related complications but not with infectious complications. Likewise, we found that htTLV ≥ 1,600 mL/m was associated with a 5-fold increased risk for pressure-related symptoms (OR 4.98, 95% CI: 1.07–23.26), but not with pain nor GI symptoms, after adjusting for age, gender, Log (htTKV), CKD stages, albumin, total cholesterol, AST, and ALP levels (Table 4). By contrast, htTKV showed no significant effect on the hepatic complications or presence of any symptoms in the multivariate analysis. Female gender was an independent risk factor for both pressure-related complications (OR 2.36, 95% CI: 1.41–3.97) and abdominal symptoms (OR 2.18, 95% CI 1.22–4.07).

**Table 3:** Multivariate binominal logistic regression analysis of hepatic complications.

Variables	Group	Subject	All complications		Pressure-related complications		Infectious complications	
		n	Univariate	* Multivariate	Univariate	* Multivariate	Univariate	* Multivariate
			OR (95% CI)	OR (95% CI)	OR (95% CI)	OR (95% CI)	OR (95% CI)	OR (95% CI)
Gender	Male	220	1.00					
	Female	241	2.01(1.31–3.08)	2.36(1.41–3.97)	2.07(1.34–3.18)	2.49(1.48–4.21)	0.91(0.37–2.47)	NS
htTLV	htTLV<1,600 mL/m	385	1.00					
	htTLV≥1,600 mL/m	76	6.02(3.56–10.18)	6.14(2.97–12.72)	6.12(3.62–10.35)	6.24(3.01–12.96)	7.38(2.66–20.51)	NS
Log (htTKV)		451	1.60(1.21–2.12)	NS	1.62(1.22–2.15)	NS	2.37(1.20–4.67)	NS

* Multivariate model was adjusted for age, gender, htTLV, Log (htTKV), CKD stages, albumin, total cholesterol, aspartate aminotransferase, and alkaline phosphatase.

OR, odds ratio; CI, confidence interval; NS, not significant; htTLV, height-adjusted total liver volume; htTKV, height-adjusted total kidney volume; CKD, chronic kidney disease.

**Table 4:** Multivariate binominal logistic regression analysis of symptoms.

Variables	Group	Subject	Pressure symptoms		Pain		GI symptoms	
			Univariate	* Multivariate	Univariate	* Multivariate	Univariate	* Multivariate
		n	OR (95% CI)	OR (95% CI)	OR (95% CI)	OR (95% CI)	OR (95% CI)	OR (95% CI)
Gender	Male	128	1.00					
	Female	125	3.03(2.02–4.55)	2.18(1.22–4.07)	1.95(1.35–2.82)	2.39(1.24–4.60)	1.60(1.12–2.30)	2.07(1.16–3.67)
htTLV	htTLV<1,600 mL/m	201	1.00					
	htTLV≥1,600 mL/m	52	7.34(3.01–1.76)	4.98(1.07–23.26)	12.00(4.33–33.28)	NS	2.47(1.35–4.49)	NS
Log (htTKV)		223	1.11(1.06–1.15)	NS	1.17(1.12–1.22)	NS	1.02(0.98–1.06)	NS

* Multivariate model was adjusted for age, gender, htTLV, Log (htTKV), CKD stages, albumin, total cholesterol, aspartate aminotransferase, and alkaline phosphatase.

GI, gastrointestinal; OR, odds ratio; NS, not significant; htTLV, height-adjusted total liver volume; htTKV, height-adjusted total kidney volume; CKD, chronic kidney disease.

### Relationship between htTLV and htTKV

We found a weak correlation between htTKV and htTLV (r^2^ = 0.062, P<0.001; Fig 3C). When all patients were divided into four groups according to htTLV of 1,600 mL/m and htTKV of 1,000 mL/m, we found a female predominance (female:male~2.5–2.6) in 9.5% of patients with htTLV. ≥1,600 mL/m and htTKV≥1,000 mL/m and in 6.2% of patients with htTLV≥1,600 mL/m and htTKV<1,000 mL/m. By contrast, we also found a male predominance (male:female~1.67) in 28.5% of patients with htTKV≥1,000 mL/m and htTLV<1,600 mL/m. Thus, 15.7% of patients displayed a female predominance for large htTLV which might be concordant or discordant with their htTKV. By contrast, 28.5% of patients displayed a male predominance for large htTKV that was discordant of their htTLV. We also found a linear correlation between height adjusted total abdominal volume (htTAV; defined as the sum of htTLV and htTKV) with htTLV (r^2^ = 0.625) (Fig 3D). However, due to patients with discordant htTLV and htTKV, the log odds of having two or more pressure–related symptoms displayed a variable and non-linear relationship with htTAV (S3 Fig).

## Discussion

Liver cysts are highly prevalent and occurred in more than 90% in our patients by 40 years of age. Temmerman et al. reported that 75% and 91% male and female ADPKD patients 25–34 years of age had liver cysts [6], which was similar to the present study. In our study, female under 40 years of age showed a higher prevalence of liver cysts suggesting that they may develop earlier in females. Although there was no significant gender difference in the median htTLV, we found a skewed distribution towards larger htTLV in women and a female predominance of moderate (htTLV ≥1,600 mL/m) and severe (htTLV≥3,200 mL/m) PLD. Notably, most (95.5%) of our 22 patients with severe PLD were female. While female sex hormones may play a role [17–19], additional as yet unidentified genetic and/or environmental factors are likely responsible for these extreme cases of PLD which could be concordant or discordant with their PKD. In contrast to our findings in PLD, we found a male predominance of our severe cases of PKD which were typically discordant to their PLD.

Analogous to TKV which predicts renal disease progression and complications [20–22], htTLV is highly associated with liver cyst-related complications and abdominal symptoms. We found that the probability of having two or more symptoms increases constantly as the liver volume expands. Moderate to severe PLD, which represents ~15% of all subjects, was associated with pressure-related symptoms and complications in our multivariate analyses. Bilateral leg edema was the most common complication and was more prevalent in the moderate to severe PLD group, even among patients with eGFR≥30 mL•min^−1^•1.73 m^−2^. By contrast, we did not find any association between htTLV and infectious complication in the multivariate model and 7 of 16 infection cases had htTLV < 1,600 mL/m. Thus, liver cyst infection appeared to be independent of the size of the cystic liver [17].

Enlarged kidneys can contribute to abdominal symptoms. Suwabe et al. demonstrated that htTAV was correlated with abdominal distention, but not with quality of life using the SF-36 [23]. We believe htTLV is a better marker for “mass-effect” complications and abdominal symptoms instead of htTAV or htTKV for the following reasons:i) htTKV showed no relationship to abdominal symptoms and hepatic complications in multivariate analysis; ii) htTLV showed a linear relationship to htTAV; iii) the log odds of having two or more pressure-related symptoms was not linear with htTAV; iv) the odds ratios (OR) for pressure-related symptoms was higher when using htTLV (OR 4.98, 95% CI 1.07–23.26) than htTAV (OR 3.62, 95% CI 1.44–9.11), indicating stronger predictive power of htTLV. In terms of quality of life, Wijnards et al. found no correlation between the TLV and health-related quality of life (HRQoL) in 92 patients with ADPKD and PLD [24]. Though the quality of life was not investigated directly, results obtained from scoring of symptoms reflected possible association between htTLV and HRQoL, This discrepancy may be attributable to the difference in study populations. The subjects in the study by Wijnards was more female predominant (89.1% vs. 75%), younger (mean age: 50 vs. 56 years), and had much larger livers (mean htTLV: 4,906 vs 2,119 mL/m), compared to the present study.

Severe PLD with significant “mass effect” is a significant cause of morbidity [6]. In our multivariate analyses we found a significant risk (OR 6.15, 95% CI 2.97–12.72) of hepatic complications is associated with htTLV≥1,600 mL/m and this risk is further increased with htTLV≥2,100 mL/m. Similarly, we found also a significant risk (OR 4.98, 95% CI 1.07–23.26) of pressure related symptoms is increased with htTLV≥1,600 mL/m. Moreover, the htTLV of our patients who required therapeutic interventional ranged from 1,803 to 13,412 mL/m. Thus, htTLV of 1,600 mL/m as the cut-off value for moderate PLD is clinically relevant. Of note, this threshold cutoff corresponds to severe PLD in another recent study [7].

This study has several limitations. This is a cross-sectional study with some gaps between the ascertainment of complications and CT scan, up to one year. However, this is unlikely to significantly affect our results as annual liver cyst growth rates range from 0.92–3.2% [25–27]. Not all patients assessed for hepatic complications had completed their questionnaire for abdominal symptoms. However, the clinical characteristics of those who completed the research questionnaire were very similar to those who did not; thus, the sub-group of patients studied for their abdominal symptoms are likely representative of the entire cohort. Scoring of abdominal symptoms was subjective and semi-quantitative but they were applied in a standardized manner. Finally, underlying diseases such as gastroesophageal reflux disease or peptic ulcer would not be accounted for in the symptom analysis.

In summary, we found the significant correlation between liver volume and PLD-related complications and symptoms in a large cohort of ADPKD from a single center. Our data demonstrated that symptoms and complications related to liver cysts increase in patients with moderate to severe PLD, and therapeutic interventions were required in a majority of cases with severe PLD. Our study defines htTLV≥1,600 mL/m as a clinical relevant threshold for moderate PLD. Patients with moderate PLD based on their symptoms or htTLV should be referred to centers with special expertise for timely interventions to manage their “mass-effect”. Mechanistic studies to delineate the basis of female predominance in moderate to severe PLD may provide novel therapeutic targets for this important clinical problem.

## Supporting Information

S1 Fig Study patient selection. (TIF)

S2 Fig Prevalence of hepatic complications and mean scores of abdominal symptoms according to height-adjusted total liver volume (htTLV) groups. (A) shows increasing prevalence of complications according to the severity of polcystic liver disease (PLD). Pressure-related and infectious complications showed significantly higher prevalence as the htTLV groups went higher with linear by linear association test (Chi-square test for trend), P<0.001; no or mild PLD, htTLV <1,600 mL/m; moderate PLD, 1,600≤ htTLV <3,200 mL/m; and severe PLD, htTLV≥3,200 mL/m. (B) The mean scores of symptoms increased in all three categories as the htTLV groups went higher with Jonckheere–Terpstra test (P for trend of non-parametric tests), P<0.001.
(TIF)

S3 Fig Likelihood (log odds) of having two or more pressure-related symptoms according to height-adjusted total liver volume (htTLV) plus total kidney volume (htTKV). (TIF)

S1 Table Baseline characteristics of two cohorts, size and complication assessment and a subset of them with additional clinical symptom assessment. (DOCX)

S2 Table Clinical characteristics of patients according to no or mild, moderate, and severe PLD. (DOCX)

S3 Table Symptom questionnaire. (DOCX)
