# Supplementary figures and images for: Clinical Correlates of Mass Effect in Autosomal Dominant Polycystic Kidney Disease
Source: PLoS One. 2015 Dec 7;10(12):e0144526. doi: 10.1371/journal.pone.0144526 (PMC4671651; doi:10.1371/journal.pone.0144526)

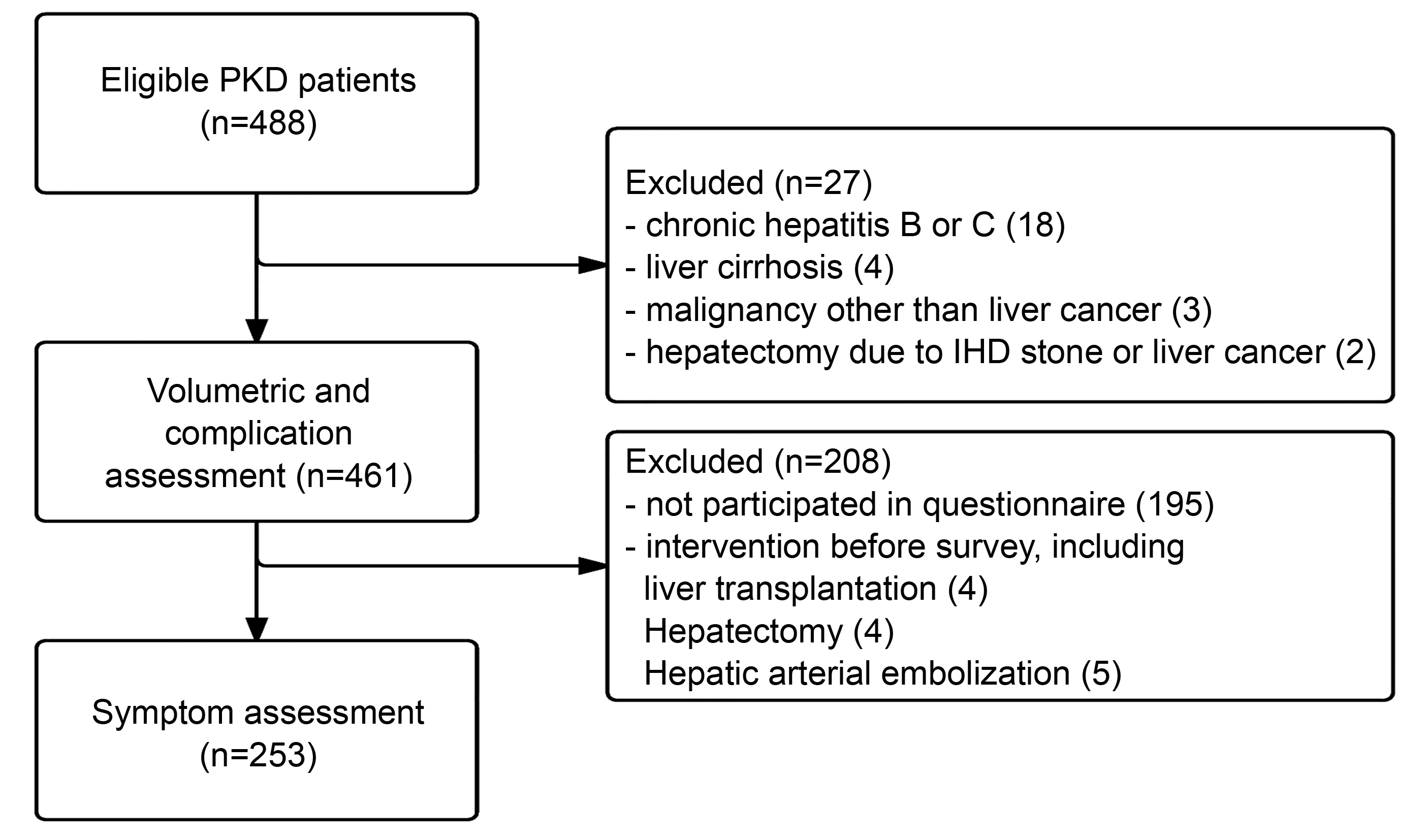

Supplement: S1 Fig — (TIF) [file pone.0144526.s001.tif]

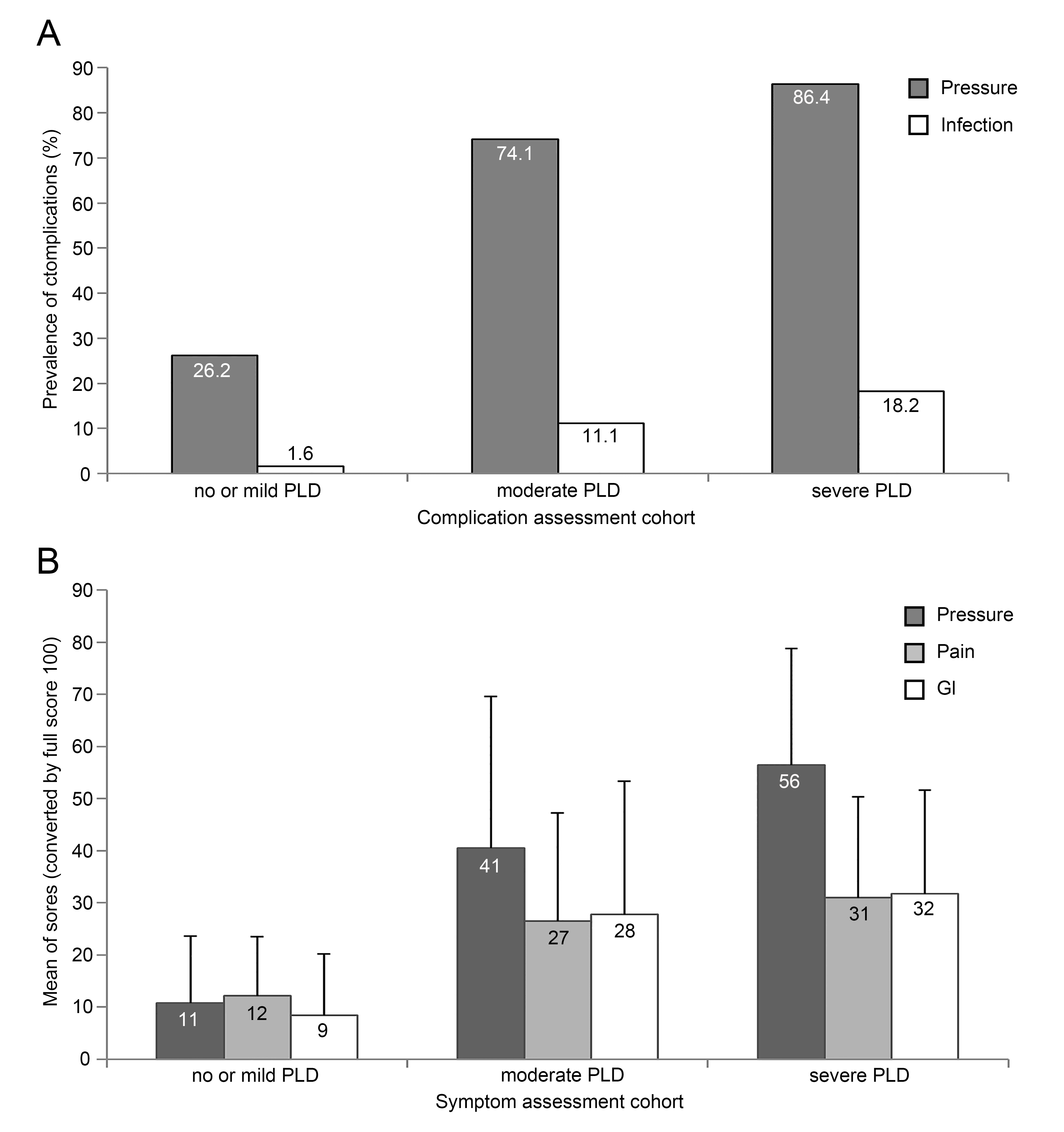

Supplement: S2 Fig — (A) shows increasing prevalence of complications according to the severity of polcystic liver disease (PLD). Pressure-related and infectious complications showed significantly higher prevalence as the htTLV groups went higher with linear by linear association test (Chi-square test for trend), P<0.001; no or mild PLD, htTLV <1,600 mL/m; moderate PLD, 1,600≤ htTLV <3,200 mL/m; and severe PLD, htTLV≥3,200 mL/m. (B) The mean scores of symptoms increased in all three categories as the htTLV groups went higher with Jonckheere–Terpstra test (P for trend of non-parametric tests), P<0.001. (TIF) [file pone.0144526.s002.tif]

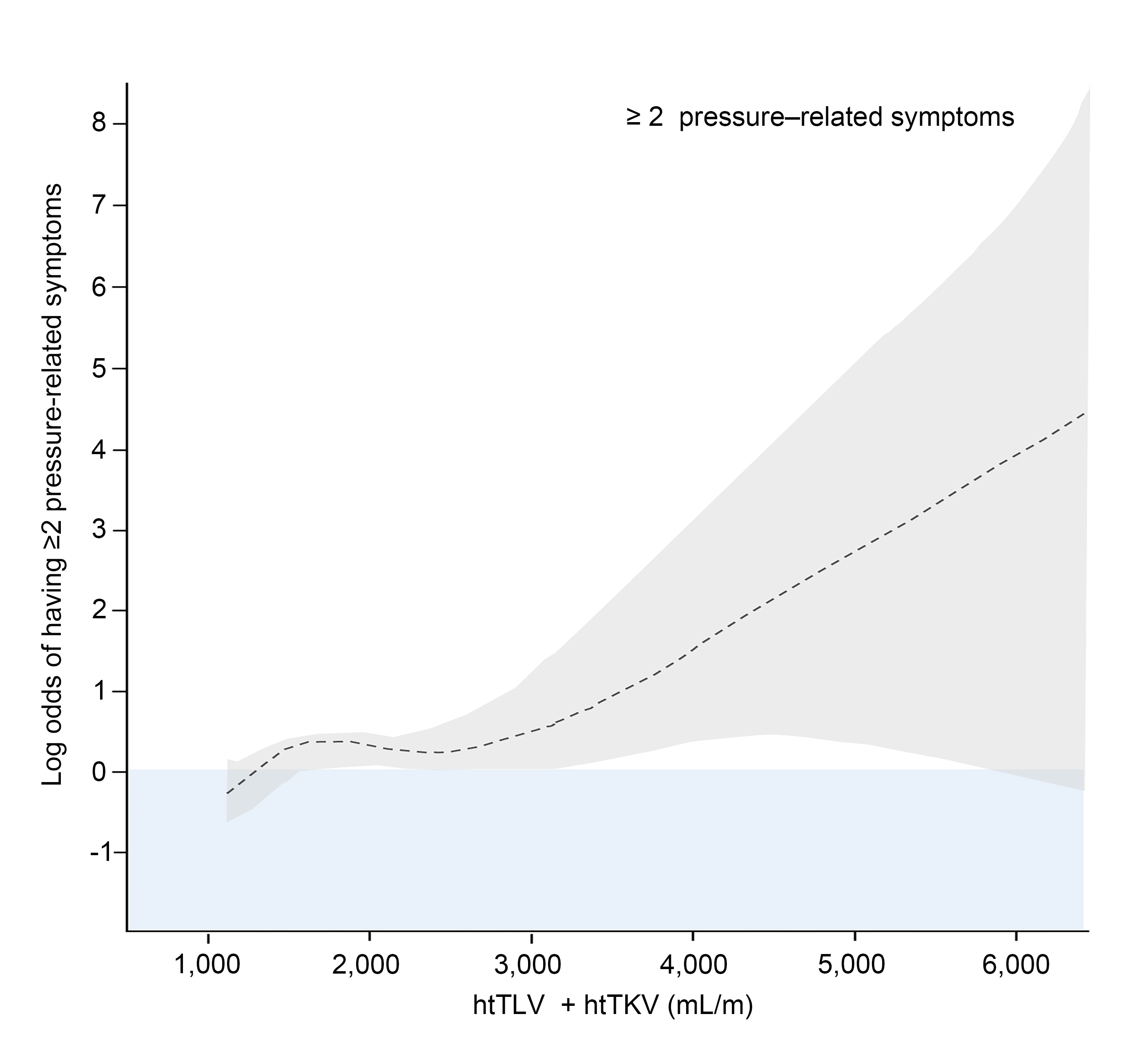

Supplement: S3 Fig — (TIF) [file pone.0144526.s003.tif]
