# Supplementary material for: Clinical Correlates of Mass Effect in Autosomal Dominant Polycystic Kidney Disease
Source: PLoS One. 2015 Dec 7;10(12):e0144526. doi: 10.1371/journal.pone.0144526 (PMC4671651; doi:10.1371/journal.pone.0144526)
Supplement: S1 Table — (DOCX) [file pone.0144526.s004.docx]

S1 Table. Baseline characteristics of two cohorts, size and complication assessment and a subset of them with additional clinical symptom assessment

|  | | Patients assessed for kidney and liver volumes (KLV) and mass-effect complications (MEC)  (n=461) | Patients assessed for KLV, MEC and clinical symptoms  (n=253) | P value ^a^ |
| --- | --- | --- | --- | --- |
| Age [mean ± SD] | | 51.3 ± 12.6 | 50.2 ± 13.0 | 0.068 |
| Female [n (%)] | | 241 (52.3) | 125 (49.4) | 0.190 |
| eGFR^*^(CKD-EPI), mL•min^−1^•1.73 m^−2^, [mean ± SD] | | 80.0 ± 27.7 | 70.1 ± 27.7 | 0.165 |
| CKD stages | Stage 1 | 101 (22.7) | 59 (23.9) | 0.001 |
|  | Stage 2 | 132 (29.7) | 71 (28.7) |  |
|  | Stage 3 | 94 (21.1) | 70 (28.3) |  |
|  | Stage 4 | 24 (5.4) | 12 (4.9) |  |
|  | Stage 5 | 94 (21.1) | 35 (14.2) |  |
| Hypertension [n(%)] | | 365 (79.2) | 204 (80.6) | 0.421 |
| htTKV [median (IQR)] | | 820 (453, 1345) | 847 (461, 1444) | 0.481 |
| htTLV [median (IQR)] | | 986 (825, 1280) | 989 (840, 1342) | 0.434 |

^a^ with linear by linear association test (Chi-square test for trend) or ; *excluded renal replacement therapy or kidney transplantation (94 cases). eGFR (CKD-EPI), estimated glomerular filtration rate (Chronic Kidney Disease-Epidemiology); CKD, chronic kidney disease; htTLV, height-adjusted total liver volume; IQR, inter-quartile range; htTKV, height-adjusted total kidney volume.
