# Supplementary material for: Clinical Correlates of Mass Effect in Autosomal Dominant Polycystic Kidney Disease
Source: PLoS One. 2015 Dec 7;10(12):e0144526. doi: 10.1371/journal.pone.0144526 (PMC4671651; doi:10.1371/journal.pone.0144526)
Supplement: S2 Table — (DOCX) [file pone.0144526.s005.docx]

S2 Table. Clinical characteristics of patients according to no or mild, moderate, and severe PLD

|  | | No or mild | Moderate | Severe | P value ^a^ |
| --- | --- | --- | --- | --- | --- |
| n(%) | | 385 (83.5) | 54 (11.7) | 22 (4.8) |  |
| Female [n(%)] | | 187 (48.6) | 33 (61.1) | 21(95.5) | <0.001 |
| Age, yr [mean ± SD] | | 50.2 ± 13.0 | 57.8 ± 8.4 | 54.9 ± 7.7 | <0.001 |
| htTLV, mL/m [median(IQR)] | | 942 (807, 1098) | 2048 (1769, 2699) | 4036 (3613, 5501) | <0.001 |
| htTKV, mL/m [median(IQR)] | | 721 (408, 1265) | 1281 (835, 1971) | 1338 (871, 2151) | <0.001 |
| HTN [n(%)] | | 295 (76.6) | 50 (92.6) | 20 (90.9) | <0.001 |
| eGFR^*^(CKD-EPI), mL•min^−1^•1.73m^−2^, [mean ± SD] | | 74 ± 28 | 55 ± 21 | 63 ± 24 | <0.001 |
| CKD stage | Stage 1 | 98 (26.1) | 2 (4.0) | 1 (5.0) | <0.001 |
|  | Stage 2 | 117 (31.2) | 11 (22.0) | 4 (20.0) |  |
|  | Stage 3 | 72 (19.2) | 16 (32.0) | 6 (30.0) |  |
|  | Stage 4 | 22 (5.9) | 2 (4.0) | 0 (0) |  |
|  | Stage 5 | 66 (17.6) | 19 (38.0) | 9 (45.0) |  |
| Intervention**^b^** [n(%)] | | 0 (0) | 7 (13.0) | 8 (36.4) | <0.001 |
| Albumin, mg/dL [mean ± SD] | | 4.3 ± 0.3 | 4.0 ± 0.5 | 3.8 ± 0.6 | <0.001 |
| TC, mg/dL [mean ± SD] | | 178 ± 29 | 160 ±33 | 155 ± 31 | <0.001 |

^a^ with linear by linear association test (Chi-square test for trend) or Jonckheere–Terpstra test (non-parametric tests for trend) as appropriate; No or mild PLD, htTLV<1,600 mL/m; Moderate PLD, 1,600≤ htTLV <3,200mL/m; Severe PLD, htTLV≥3,200 mL/m; * excluded RRT or KT (94 cases). ^b^ Liver transplantation, partial hepatectomy or transcatheter arterial embolization (TAE). htTLV, height-adjusted total liver volume; PLD, polycystic liver disease; htTKV, height-adjusted total kidney volume; HTN, hypertension; eGFR (CKD-EPI), estimated glomerular filtration rate (Chronic kidney disease epidemiology); TC, total cholesterol.
