# Supplementary material for: Clinical Correlates of Mass Effect in Autosomal Dominant Polycystic Kidney Disease
Source: PLoS One. 2015 Dec 7;10(12):e0144526. doi: 10.1371/journal.pone.0144526 (PMC4671651; doi:10.1371/journal.pone.0144526)
Supplement: S3 Table — (DOCX) [file pone.0144526.s006.docx]

S3 Table. Symptom questionnaire

| Category | Abdominal  symptoms | Grading of symptoms | score |
| --- | --- | --- | --- |
| Pressure | Early satiety | 0 – No or transient events | 3 |
|  |  | 1 – Occasional events interfering with some social activities |  |
|  |  | 2 – Prolonged and troublesome events causing requests for relief and interfering with many social activities |  |
|  |  | 3 – Severe or continuous events with impact on all social activities |  |
|  | Sense of a mass | 0 – Not palpable | 1 |
|  |  | 1 – Palpable |  |
|  | Dyspnea or chest discomfort | 0 – No or transient events | 3 |
|  |  | 1 – Occasional events interfering with some social activities |  |
|  |  | 2 – Prolonged and troublesome events causing requests for relief and interfering with many social activities |  |
|  |  | 3 – Severe or continuous events with impact on all social activities |  |
|  | Abdominal distention | 0 – No or transient events | 3 |
|  |  | 1 – Occasional events interfering with some social activities |  |
|  |  | 2 – Prolonged and troublesome events causing requests for relief and interfering with many social activities |  |
|  |  | 3 – Severe or continuous events with impact on all social activities |  |
| Pain | Right upper quadrant pain or discomfort | 0 – No or transient events | 3 |
|  |  | 1 – Occasional events interfering with some social activities |  |
|  |  | 2 – Prolonged and troublesome events causing requests for relief and interfering with many social activities |  |
|  |  | 3 – Severe or continuous events with impact on all social activities |  |
|  | Flank pain or discomfort | 0 – No or transient events | 3 |
|  |  | 1 – Occasional events interfering with some social activities |  |
|  |  | 2 – Prolonged and troublesome events causing requests for relief and interfering with many social activities |  |
|  |  | 3 – Severe or continuous events with impact on all social activities |  |
|  | Back pain or discomfort | 0 – No or transient events | 3 |
|  |  | 1 – Occasional events interfering with some social activities |  |
|  |  | 2 – Prolonged and troublesome events causing requests for relief and interfering with many social activities |  |
|  |  | 3 – Severe or continuous events with impact on all social activities |  |
|  | Taking analgesics | 0 – None | 3 |
|  |  | 1 – Occasional |  |
|  |  | 2 – Once a week |  |
|  |  | 3 – Almost every day |  |
| GI | Anorexia | 0 – No or transient events | 3 |
|  |  | 1 – Occasional events interfering with some social activities |  |
|  |  | 2 – Prolonged and troublesome events causing requests for relief and interfering with many social activities |  |
|  |  | 3 – Severe or continuous events with impact on all social activities |  |
|  | Nausea or vomiting | 0 – None | 3 |
|  |  | 1 – Occasional events interfering with some social activities |  |
|  |  | 2– Frequent nausea, no vomiting |  |
|  |  | 3 – Nausea almost every day and frequent vomiting |  |
|  | Epigastric soreness | 0 – No or transient events | 3 |
|  |  | 1 – Occasional events interfering with some social activities |  |
|  |  | 2 – Prolonged and troublesome events causing requests for relief and interfering with many social activities |  |
|  |  | 3 – Severe or continuous events with impact on all social activities |  |
| Total |  | | 31 |

* This questionnaire was modified from Gastrointestinal Symptom Rating Scale questionnaire (15) and written in Korean. GI, Gastrointestinal.
